# [^18^F]FDG PET/CT for evaluating early response to neoadjuvant chemotherapy in pediatric patients with sarcoma: a prospective single-center trial

**DOI:** 10.1186/s13550-020-00715-0

**Published:** 2020-10-15

**Authors:** Giulia Polverari, Francesco Ceci, Roberto Passera, Jacquelyn Crane, Lin Du, Gang Li, Stefano Fanti, Nicholas Bernthal, Fritz C. Eilber, Martin Allen-Auerbach, Johannes Czernin, Jeremie Calais, Noah Federman

**Affiliations:** 1 Ahmanson Translational Theranostics Division, Department of Molecular and Medical Pharmacology, David Geffen School of Medicine, University of California Los Angeles, 200 Medical Plaza, Suite B114-61, Los Angeles, CA 90095 USA; 2 Nuclear Medicine, S.Orsola-Malpighi University Hospital, University of Bologna, Bologna, Italy; 3 PET/CT Center, Affidea IRMET, Turin, Italy; 4 Nuclear Medicine, Department of Medical Sciences, University of Turin, Turin, Italy; 5 Department of Pediatrics, David Geffen School of Medicine, University of California Los Angeles, Los Angeles, CA USA; 6 Department of Biostatistics, Jonathan and Karin Fielding of Public Health, University of California at Los Angeles, Los Angeles, CA USA; 7 Department of Orthopedics, David Geffen School of Medicine, University of California Los Angeles, Los Angeles, CA USA; 8 Division of Surgical Oncology, David Geffen School of Medicine, University of California Los Angeles, Los Angeles, CA USA; 9 Jonsson Comprehensive Cancer Center, University of California Los Angeles, Los Angeles, CA USA

**Keywords:** [^18^F]FDG, PET/CT, Sarcoma, Neoadjuvant chemotherapy, Therapy response, Pediatrics

## Abstract

**Introduction:**

This is a prospective, single-center trial in pediatric patients with sarcoma aiming to evaluate [^18^F]FDG PET/CT as a tool for early response assessment to neoadjuvant chemotherapy (neo-CTX).

**Methods:**

Bone or soft tissue sarcoma patients with (1) baseline [^18^F]FDG PET/CT within 4 weeks prior to the start of neo-CTX (PET1), (2) early interim [^18^F]FDG PET/CT (6 weeks after the start of neo-CTX (PET2), (3) evaluation of neo-CTX response by histology or MRI, and (4) definitive therapy after neo-CTX (surgery or radiation) were included. Semiquantitative PET parameters (SUVmax, SUVmean, SUVpeak, MTV and TLG) and their changes from PET1 to PET2 (ΔPET) were obtained. The primary endpoint was to evaluate the predictive value of PET1, PET2 and ΔPET parameters for overall survival (OS) and time to progression (TTP). The secondary outcome was to evaluate if [^18^F]FDG PET/CT can predict the response to neo-CTX assessed by histopathology or MRI. Primary and secondary outcomes were also evaluated in a subpopulation of patients with bone involvement only.

**Results:**

Thirty-four consecutive patients were enrolled (10 females; 24 males; median age 15.1 years). 17/34 patients (50%) had osteosarcoma, 13/34 (38%) Ewing's sarcoma, 2/34 (6%) synovial sarcoma and 2/34 (6%) embryonal liver sarcoma. Median follow-up was 39 months (range 16–84). Eight of 34 patients (24%) died, 9/34 (27%) were alive with disease, and 17/34 (50%) had no evidence of residual/recurrent disease. Fifteen of 34 (44%) and 19/34 (56%) were responders and non-responders, respectively. PET2-parameters were associated with longer TTP (*p* < 0.02). ΔMTV was associated with tissue response to neo-CTX (*p* = 0.047). None of the PET1, PET2 or ΔPET parameters were associated with OS.

**Conclusion:**

[^18^F]FDG PET/CT performed 6 weeks after the start of neo-CTX can serve as an early interim biomarker for TTP and pathologic response but not for OS in pediatric patients with sarcoma.

## Introduction

Bone and soft tissue sarcomas are the most common primary bone malignancies in children. Their incidence ranges from 0.2 to 0.3/100,000/year [1, 2]. Depending on the histologic sarcoma subtype, patient management may include neo-adjuvant chemotherapy (neo-CTX) and radical surgery with or without radiation therapy (RT), followed by adjuvant CTX. For soft tissue sarcomas, the therapeutic strategy is based on a risk classification that considers TNM stage, histologic subtype, and primary tumor location. Local recurrence or distant metastases occur in up to 40% of patients who initially receive treatment with curative intent [3]. The 5-year survival rate in patients with metastases is 20% compared to 65% for patients with localized disease [4]. Clinical characteristics such as tumor grade, size, presence of distant metastases or skip lesions, surgical margin status and histologic response to neo-CTX have been reported to be predictors of survival in bone and soft tissue sarcomas [5–7]. However, these prognostic factors are not highly accurate. Histologic response is determined by examination of resected specimens after the completion of neo-CTX. A noninvasive early interim biomarker that could permit reliable response predictions would be useful to guide changes in treatment of non-responding patients [8].

[^18^F]FDG PET/CT is used to accurately stage and assess treatment response in almost all cancers, including those in pediatric patients [9–12]. However, for pediatric bone and soft tissue sarcoma, the role of [^18^F]FDG PET/CT is not clearly defined. In this prospective study, we assessed the value of semiquantitative [^18^F]FDG PET/CT parameters as a potential early intermediate biomarker for response to neo-CTX in pediatric patients with high-grade bone and soft tissue sarcomas.

## Methods

### Objectives

The primary aim of this prospective study was to evaluate whether semiquantitative [^18^F]FDG PET/CT parameters acquired at baseline (PET1) and during therapy (PET2) are predictive of time to progression (TTP) and overall survival (OS) in children with high-grade bone or soft tissue sarcomas.

The secondary objective was to determine whether [^18^F]FDG PET/CT was able to predict the response to neo-CTX defined by percent tumor necrosis in the resected tumor or by MRI performed after the completion of neo-CTX. Primary and secondary aims were also evaluated in the subpopulation of patients with bone sarcomas.

### Study design and participants

This was a prospective, open-label, observational, single-arm, single-center study approved by the local ethics committee (UCLA-IRB#10-000246) in pediatric patients with high-grade bone or soft tissue sarcomas. All patients with histologically or cytologically confirmed bone or soft tissue sarcoma who were evaluated for management of disease prior to neo-CTX before definitive therapy (surgery, radiation therapy (RT)) were eligible. Written informed consent was obtained from all participants or guardians at enrollment along with signed participant assent, when applicable.

Enrolled patients underwent [^18^F]FDG PET/CT at 2 time points: within 4 weeks before the start of neo-CTX (baseline, PET1) and at 6 weeks after the start of CTX (early interim, PET2).

[^18^F]FDG PET/CT findings were confirmed by pathology when available or by follow-up [^18^F]FDG PET/CT and/or standard clinical follow-up.

Excised tumors were examined for extent of necrosis, and ≥ 90% necrosis (< 10% viable tumor cells) was considered a complete histopathological response to neo-CTX [13]. In patients undergoing definitive RT, response was assessed by MRI at the end of neo-CTX [14, 15]. Patients with a complete disappearance of the soft tissue component of the tumor on MRI were considered responders. Pathology and MRI clinical reports were used to obtain the pathological response.

### [^18^F]FDG PET/CT image acquisition

Patients were instructed to fast for at least 6 h before the scan, and blood glucose levels were measured before injection of [^18^F]FDG. All patients had serum glucose levels of < 150 mg/dl prior to the scan. None of the patients had a history of diabetes.

[^18^F]FDG was administered by intravenous injection at the activity of 0.1 mCi/Kg and up to a total maximum of 10 mCi. After 60 min of uptake time, images were acquired using a 64-detector PET/CT scanner (2007 Biograph 64 Truepoint or 2010 Biograph mCT 64; Siemens). A low-dose CT for attenuation correction (132 kVp, 35 mAs (CareDose protocol), 0.5-s tube rotation, 5-mm slice collimation, bed speed 8 mm/s) was performed after administration of intravenous contrast (115 mL of iohexol [Omnipaque 350; GE Healthcare]) unless contraindicated. CT images were acquired along the same length of the patient's body as the PET (full-body PET/CT, from vertex to toes). The time per bed position was 2 min. Iterative methods were used to reconstruct the PET images with a slice thickness of 2 mm. All PET images were reconstructed using attenuation, dead-time, random-event and scatter corrections. PET images were reconstructed with an iterative algorithm (ordered-subset expectation maximization) in a 200 × 200 matrix (3-dimensional, 2 iterations, 24 subsets, Gaussian filter 5.0).

### Visual analysis

PET/CT images were retrospectively analyzed on an OsiriX workstation by two UCLA investigators (GP, FC), with more than 5 years of experience in reading oncologic PET images. The readers had access to all patient medical information. Images were interpreted by consensus. Any focal non-physiologic [^18^F]FDG uptake above surrounding background activity was considered consistent with malignancy. Metastatic sites were classified as regional lymph nodes (LNs), lung, or other distant metastases (other skeletal segments and/or other distant sites).

### Semiquantitative analysis

Standardized uptake value (SUV) was defined as activity concentration (Bq/mL) divided by injected activity (Bq) normalized to body weight. The highest voxel value (SUVmax) was obtained in a volume of interest (VOI) covering the entire tumor as defined by the investigator (GP). SUVpeak and SUVmean were also calculated within the same VOI. The metabolic tumor volume (MTV) was determined with a threshold of 40% of the SUVmax. When normal tissues with high [^18^F]FDG uptake were included in the VOIs or the VOIs excluded obvious tumor tissue, manual adjustment was applied. Total lesion glycolysis (TLG) was defined as the product of SUVmean and MTV. Reduction in SUV parameters was defined as ΔSUV = [(SUV2 − SUV1)/SUV1]. Change in MTV and TLG was calculated as follows: ΔMTV = [(MTV2 − MTV1)/MTV1]; ΔTLG = [(TLG2 − TLG1)/TLG1].

Finally, response to neo-CTX was evaluated applying PET EORTC criteria [16].

### Statistical analysis

Median and interquartile range (IQR) were used as descriptive statistics for continuous variables, while absolute and relative frequencies for categorical ones. For time-to-event data, the endpoints were: OS defined as the time interval from the start of neo-CTX to the date of last follow-up or the date of death from any cause. TTP was defined as the time interval from the start of neo-CTX to the date of the first event or the date of last follow-up for patients who had no events (recurrent or progressive disease and death from any cause).

A univariate Cox proportional hazards regression model was used to assess the association between TTP, OS and the following covariates of interest: PET1-SUVmax, PET1-SUVmean, PET1-SUVpeak, PET1-MTV, PET1-TLG, PET2-SUVmax, PET2-SUVmean, PET2-SUVpeak, PET2-MTV, PET2-TLG and changes therein. Changes between PET1 and PET2 parameters (ΔPET parameters) were expressed in percentage of reduction. Kaplan–Meier curves and log-rank test were used to summarize and compare the survival experience between PET1, PET2 and ΔPET parameters on cutoff values (median, upper quantile and lower quantile). Mann–Whitney test was used to test the association between PET1, PET2 and ΔPET parameters to the neo-CTX response status (yes/no). Data were analyzed by R 3.6.1 (R Foundation for Statistical Computing, Vienna-A, https://www.R-project.org).

## Results

### Population characteristics

**Fig. 1:**
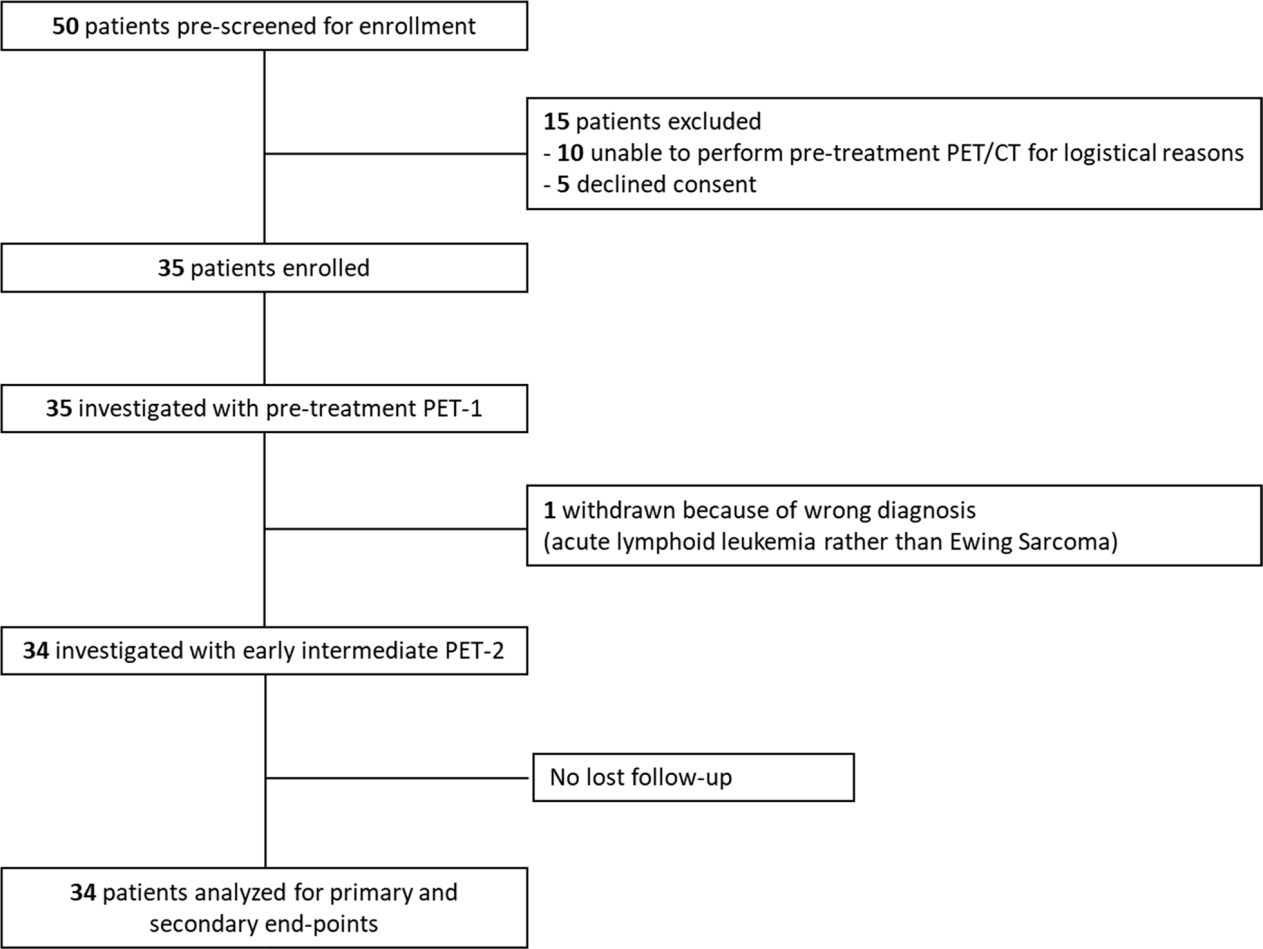
Study flowchart

The study flowchart is provided in Fig. 1, and patients characteristics are listed in Table 1. Thirty-four consecutive patients (10 females and 24 males; median age of 15.1 years; 7.4–19.7 years) were enrolled from July 2010 to November 2016: 17/34 (50%) had osteosarcoma, 13/34 (38%) had Ewing's sarcoma [4/13 (31%) soft tissue and 9/13 (69%) bone], 2/34 (6%) had synovial sarcoma, and 2/34 (6%) had embryonal sarcoma of the liver. According to the revised American Joint Committee on Cancer staging system (AJCC) [15], 16/34 (47%) patients were classified as stage IIb, 11/34 (32%) as stage IIa and 7/34 (21%) had skip or distant metastasis at diagnosis (stage III, IV; 1 skip lesion, 1 distant bone metastasis and 5 lung metastases).

**Table 1:** Study population characteristics

Characteristics	Value
Age	Median 15.1 (7.4–19.7)
Sex	
Male	24/34 (70.6%)
Female	10/34 (29.4%)
Histological variant	
Osteosarcoma	17/34 (50%)
Ewing's sarcoma	13/34 (38.2%)
Synovial sarcoma	2/34 (5.9%)
Liver embryonal sarcoma	2/34 (5.9%)
Site	
Extremities	22/34 (64.8%)
Scapula	2/34 (5.9%)
Spine	1/34 (2.9%)
Pelvis	2/34 (5.9%)
Chest/abdominal	3/34 (8.8%)
Lung	1/34 (2.9%)
Liver	3/34 (8.8%)
AJCC	
IIa	11/34 (32.4%)
IIb	16/34 (47.1%)
III	1/34 (2.9%)
IV	6/34 (17.6%)
Primary therapy	
Radical surgery	29/34 (85.3%)
Radiation therapy	5/34 (14.7%)

*AJCC* American Joint Commission on Cancer

### Therapy protocols

**Fig. 2:**
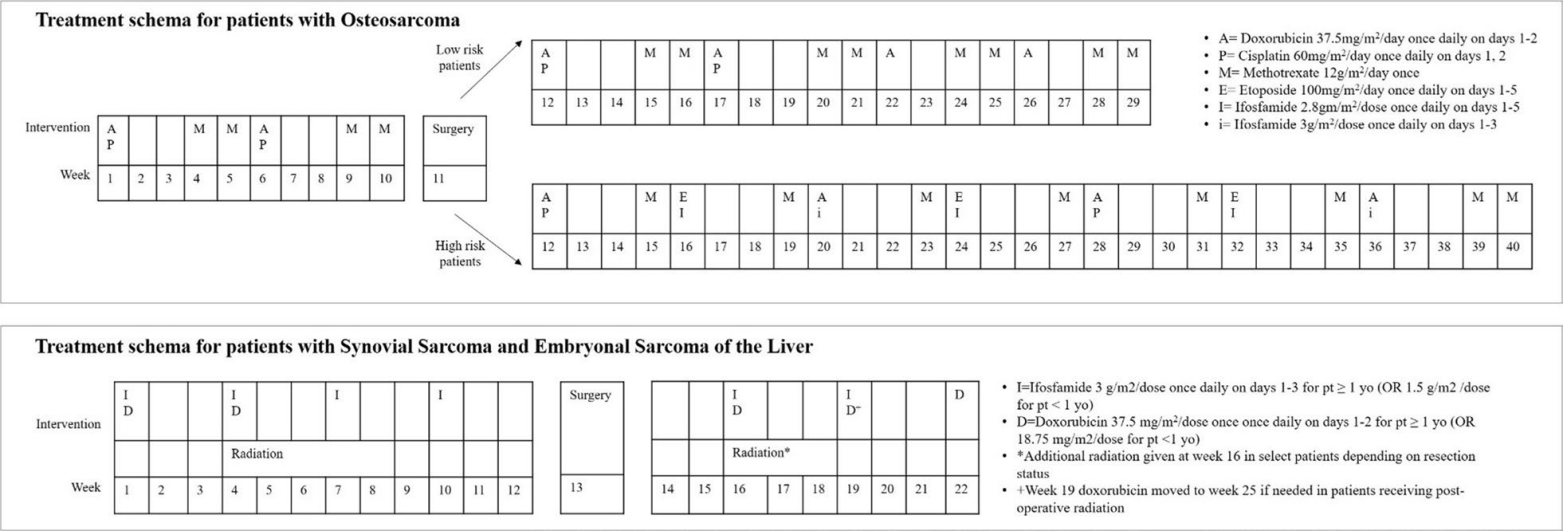
Therapy protocol

All 17 patients with osteosarcoma were treated according to the Children’s Oncology Group (COG) Protocol with 10 weeks of neo-CTX (high-dose methotrexate, doxorubicin and cisplatin) before surgery (16 resections with reconstruction/replacement, 1 amputation), followed by 18 weeks of adjuvant-CTX (high-dose methotrexate, doxorubicin and cisplatin) for low-risk patients or 29 weeks of adjuvant-CTX with addition of ifosfamide and etoposide in high-risk patients [17, 18]. Ewing's sarcoma patients (*n* = 13) were treated as per COG AEWS1031 protocol composed of an initial 12-week course of interval compression CTX (vincristine, doxorubicin, cytoxan, alternating with ifosfamide and etoposide), followed either by surgical removal of the tumor (8/13 (62%) or definitive RT (5/13 (38%)). Following definitive therapy, CTX was continued for approximately 6 months (consolidation). Patients with synovial sarcoma (*n* = 2) and embryonal sarcoma of the liver (*n* = 2) were treated with doxorubicin and ifosfamide according to COG ARST0332 combined with surgery and radiation [19]. The treatment schemas for patients with osteosarcoma, synovial sarcoma, and embryonal sarcoma of the liver are shown in Fig. 2. The treatment schema for patients with Ewing's sarcoma is not included as the data from that study are not yet published.

### Follow-up and therapy response assessment

Median follow-up was 39 months (range 16–84). 8/34 patients (24%) died from cancer-related causes, while 9/34 (27%) were alive with disease and 17/34 (50%) had no evidence of residual/recurrent disease at the last follow-up. The median time between primary therapy and the disease relapse was 14.4 months (4.2–53.4 months), and the median time between primary therapy and cancer-related death was 33.6 (19.7–73.5 months). The median OS was 71 months, while the median TTP was 33.5 months. The shortest follow-up in patient who did not show disease progression was 16 months. In 7/34 patients with metastatic disease (21%), the median OS and TTP were 35 and 13.2 months, respectively (3/7 died (43%), 4/7 were alive with disease (57%) at last follow-up).

Tumor tissue response to neo-CTX was evaluated in all patients (necrosis > 90% at histopathology of excised tumors in 29 and by MRI in 5 patients). Fifteen of 34 patients (44.1%) were classified as responders (15/15 by histopathology evaluation), while 19/34 patients (55.9%) were considered non-responders (14/34 by histopathology and 5/34 by MRI evaluation). The average percentage of CTX-induced tumor necrosis was 68%, ranging from 5 to 99%. Six deaths were reported among the non-responders (*n* = 14, median OS = 72 months), while no events were observed in the responders group (*n* = 15, median OS = not reached).

### [^18^F]FDG PET/CT findings

**Fig. 3:**
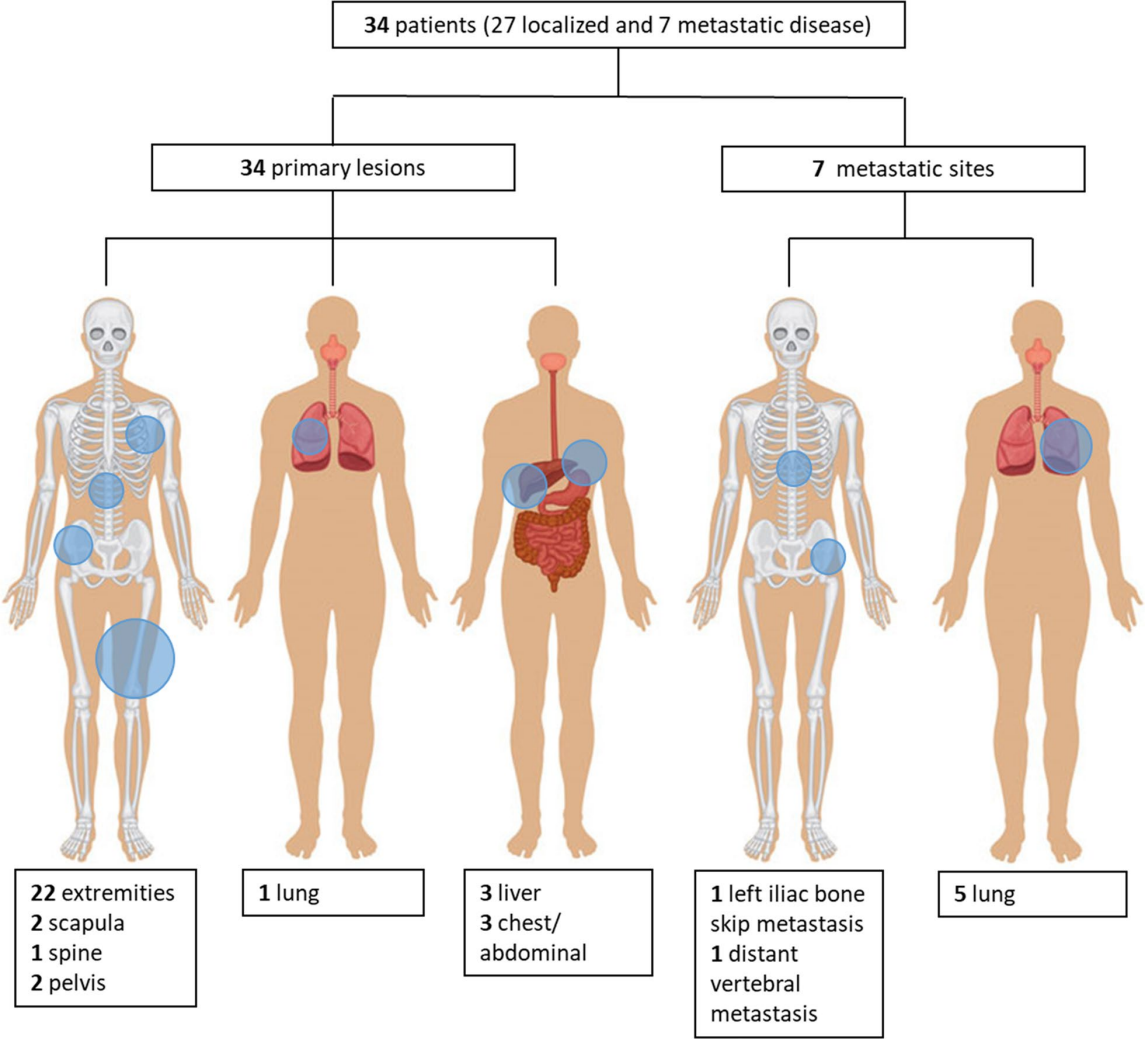
Overview of primary bone and soft tissue sarcoma localizations and metastatic sites

Primary tumors were identified on [^18^F]FDG PET/CT in all patients. In total, 27/34 patients (79%) had localized disease, while 7/34 patients (21%) had metastatic disease (Fig. 3). These included 2 patients with Ewing's sarcoma (left femur with left iliac bone metastasis; 1 patient with Ewing's sarcoma of the chest wall (11th rib) with vertebral body metastasis). Five patients (3 osteosarcomas, 1 synovial and 1 liver embryonal sarcoma) showed sub-centimeter bilateral lung nodules with faint [^18^F]FDG uptake. All metastatic lesions seen at PET1 showed partial or complete metabolic response at PET2. No new metastatic lesion at PET2 was observed.

**Fig. 4:**
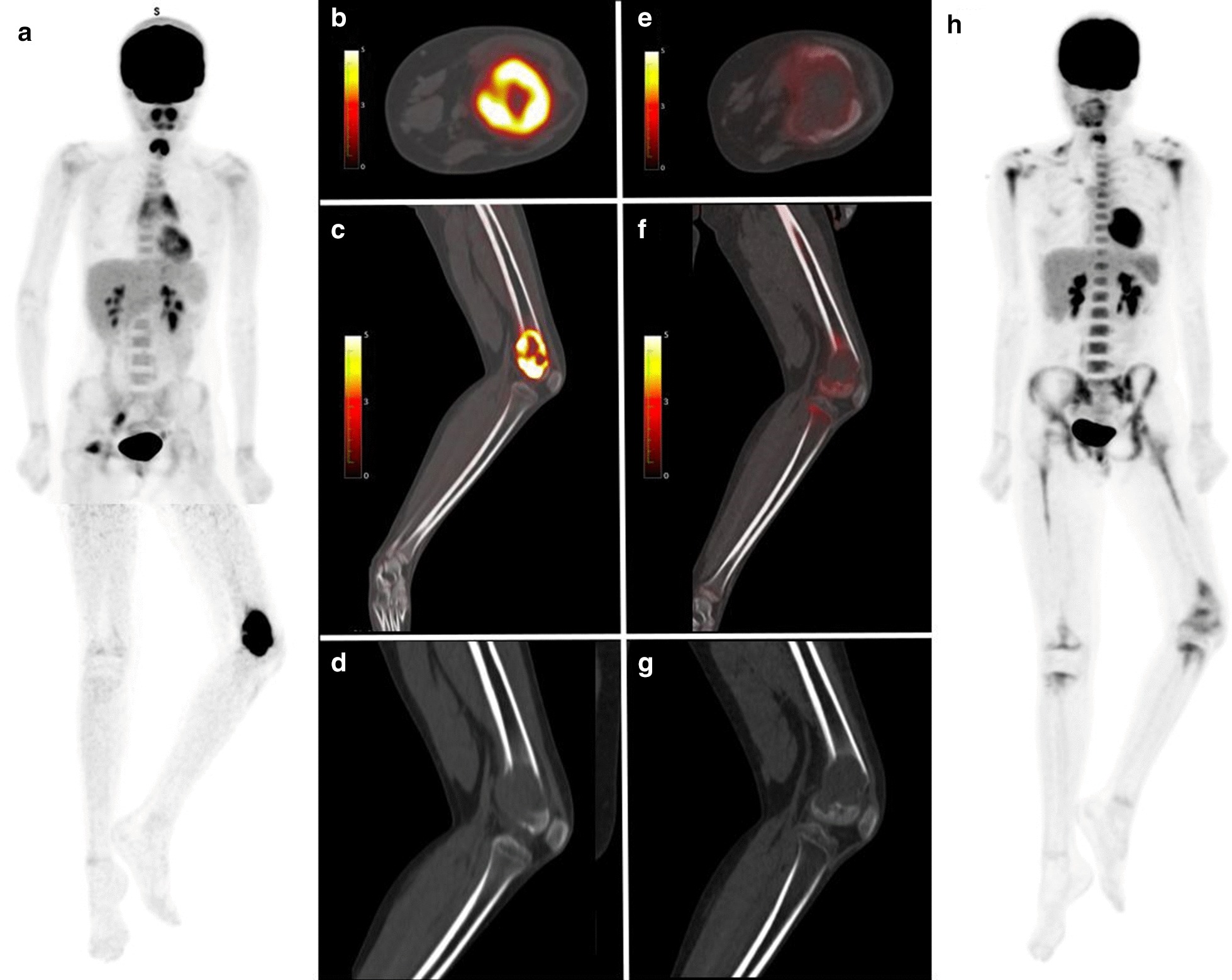
Eleven-year-old girl diagnosed with left distal femur osteosarcoma. PET-1 (**a**–**d**) showed stage IIa localized [18F]FDG avid disease (SUVmax 15.2; MTV 113.2 mm^3^; TLG 84.4). Non sarcoma related [18F]FDG uptake was visualized in the thymus (physiologic), the right adnexa (physiologic) and the right piriformis muscle (functional or strain). PET-2 (**e**–**h**) showed decreased [18F]FDG uptake (SUVmax 2.2; MTV 83.9 mm^3^; 46.1) after neo-CTX. Diffuse [18F]FDG uptake in bone marrow (**h**), was related to rebound post-CTX. Patient underwent surgery (necrosis > 99%) and had no evidence of disease (NED) at last follow-up. PET1, **a** PET 3D MIP, **b** fused PET/CT axial view, **c** fused PET/CT sagittal view, **d** CT sagittal view. PET2, **e** Fused PET/CT axial view, **f** fused PET/CT sagittal view, **g** CT sagittal view, **h** PET 3D MIP

**Fig. 5:**
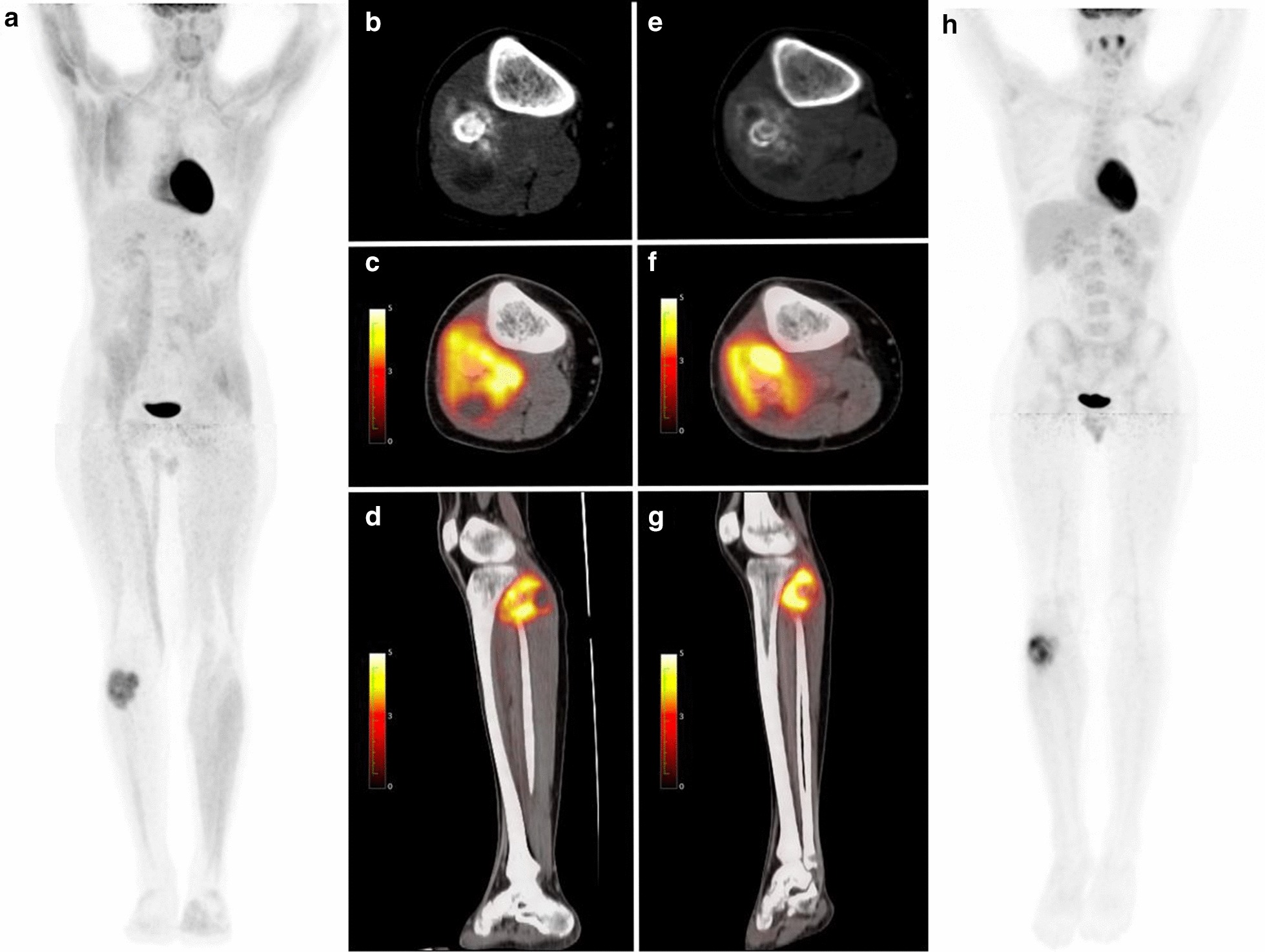
Eighteen-year-old boy diagnosed with osteosarcoma. PET-1 (**a**, **b**) showed [18F]FDG avid lesion in the right proximal fibula (SUVmax 6.6; MTV 102.2 mm^3^; TLG 91). PET-2 (**c**, **d**) did not show major [18F]FDG uptake changes after neo-CTX (SUVmax 8.6; MTV: 67.2; TLG: 80.6). Patient underwent surgery (proximal fibular and mass resection) and viable tumor was seen in the resected specimen. After completion of adjuvant CTX patient developed lung metastatic disease. Patient was alive with disease at last follow up. PET1, **a** PET 3D MIP, **b** CT axial view, **c** fused PET/CT axial view, **d** fused PET/CT sagittal view. PET2, **e** CT axial view, **f** fused PET/CT axial view, **g** fused PET/CT sagittal view, **h** PET 3D MIP

All primary tumors had increased [^18^F]FDG uptake. Baseline SUVmax, SUVmean, SUVpeak, MTV and TLG median values were 7.9 (5.3–10.6 IQR), 3.8 (3.0–4.9 IQR), 6.0 (4.0–7.4 IQR), 161.0 (85.2–262.4 IQR) and 104.7 (54.8–259.9 IQR), respectively. In PET2, the same parameters decreased to 3.1 (2.2–4.0 IQR), 2.0 (1.4–2.7 IQR), 2.4 (1.8–3.4 IQR), 72.0 (34.8–131.8 IQR) and 45.4 (19.4–105.5 IQR), respectively. PET1, PET2-parameters and their changes are listed in Table 2. Figures 4 and 5 show two examples of patients responding and non-responding to neo-CTX, respectively.

**Table 2:** [^18^F]FDG PET parameters (SUVmax, SUVmean, SUVpeak, MTV and TLG) at PET1, PET2 and their changes

PET Parameters	Minimum	Percentile 25	Median	Percentile 75	Maximum
SUVmax					
PET1	1.8	5.3	7.9	10.6	25.1
PET2	0.0	2.2	3.1	4.0	15.4
ΔPET	− 100%	− 68%	− 58%	− 29%	+ 62%
SUVmean					
PET1	0.7	3.0	3.8	4.9	7.0
PET2	0.0	1.4	2.0	2.7	6.3
ΔPET	− 100%	− 60%	− 46%	− 18%	+ 61%
SUVpeak					
PET1	0.9	4.0	6.0	7.4	21.6
PET2	0.0	1.8	2.4	3.4	10.3
ΔPET	− 100%	− 71%	− 57%	− 25%	+ 61%
MTV					
PET1	5.7	85.2	161.0	262.4	1579.9
PET2	2.6	34.8	72.0	131.8	932.3
ΔPET	− 94%	− 74%	− 44%	− 30%	+ 129%
TLG					
PET1	4	54.8	104.7	259.9	1817.3
PET2	1.8	19.4	45.4	105.5	626.1
ΔPET	− 98%	− 76%	− 58%	− 37%	+ 757%

### [^18^F]FDG PET parameters for prediction of survival

**Fig. 6:**
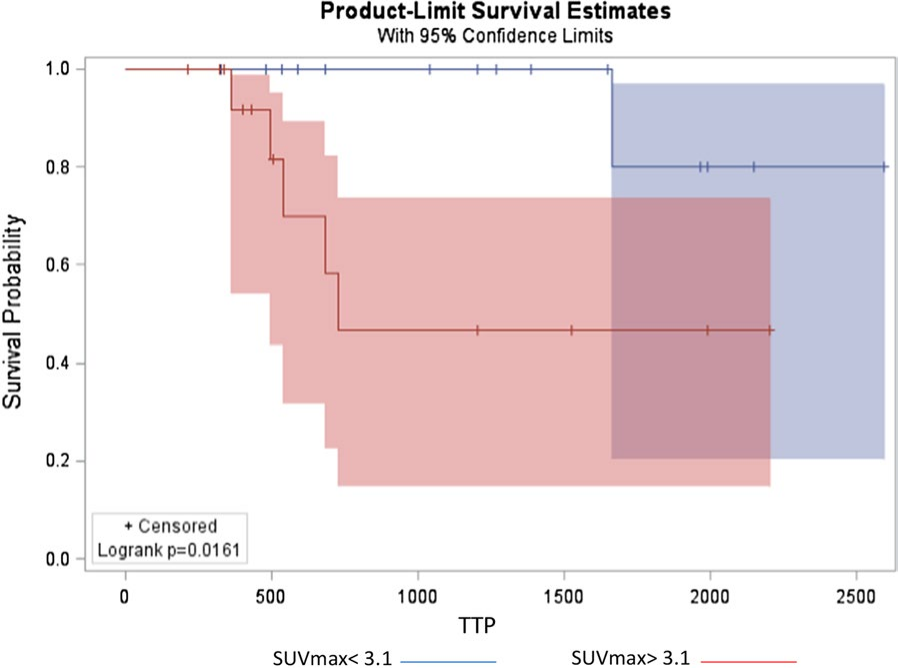
Early interim PET2-SUVmax. Kaplan–Meier plot analysis of PET2-SUVmax with TTP (*p* = 0.016). Patients were stratified by the median SUVmax = 3.1

None of PET1-parameters or the ΔPET were significantly associated with TTP in the univariate Cox regression model (Table 3). All PET2-parameters were significantly associated with TTP (Table 3, *p* < 0.02): Patients with higher SUVmax, SUVmean, SUVpeak, MTV and TLG on PET2 had earlier disease progression than those patients presenting with lower values. TTP was shorter in patients with PET2-SUVmax > 3.1 (median PET2-SUVmax) (Fig. 6, *p* = 0.016) and with PET2-SUVpeak > 2.4 (median value) (*p* = 0.02), PET2-SUVmean > 1.9 (median value) (*p* = 0.007), PET2-MTV > 131.8 (upper quartile value) (*p* = 0.01) and PET2-TLG > 105.5 (upper quartile) (*p* = 0.002). All these data are summarized in Additional file 1: Fig. S1. An association was observed for ΔSUVmean (*p* = 0.017), ΔMTV (*p* = 0.028) and ΔTLG (*p* = 0.031) with the relapse status at last follow-up (Additional file 1: Table S1). None of the PET1, PET2 and ΔPET parameters were associated with OS (Additional file 1: Table S2). Due to the low number of events (8/34 patients died, while 17/34 had a recurrent/progressive disease), multivariate Cox analysis was not performed. Finally, response to neo-CTX has been evaluated using PET parameters, applying EORTC criteria [18]. One patient was considered as complete responder according to PET EORTC criteria, 23/34 as partial responder, 4/34 as stable disease and 3/34 as progressive disease. No statistically significant association has been observed between PET EORTC response to therapy criteria and TTP (*p* = 0.59) or OS (*p* = 0.94).

**Table 3:** [^18^F]FDG PET parameters and TTP.  Univariate analysis of PET1, PET2 and ΔPET for TTP in the full study population (bone + soft tissue sarcoma)

	HR	95% CI		*p* value
		Lower	Upper	
PET1-SUVmax	1.10	0.99	1.22	0.061
PET1-SUVmean	1.15	0.86	1.53	0.346
PET1-SUVpeak	1.12	0.99	1.25	0.066
PET1-MTV	1.00	0.99	1.00	0.810
PET1-TLG	1.00	0.99	1.00	0.460
PET2-SUVmax	1.30	1.12	1.51	0.001
PET2-SUVmean	1.76	1.26	1.46	0.001
PET2-SUVpeak	1.38	1.14	1.66	0.001
PET2-MTV	1.00	1.00	1.01	0.020
PET2-TLG	1.00	1.00	1.01	0.019
ΔSUVmax	1.01	0.99	1.02	0.131
ΔSUVmean	1.01	0.99	1.02	0.123
ΔSUVpeak	1.01	0.99	1.02	0.108
ΔMTV	1.01	0.99	1.02	0.146
ΔTLG	1.00	1.00	1.01	0.127

### [^18^F]FDG PET parameters for prediction of pathological response

No significant association between PET1 and PET2-parameters and the response to neo-CTX was observed (Table 4). Despite the lack of statistical significance, the response rate was higher in patients with PET2-SUVmax < 3.1 (median value) (60% vs 21%; *p* > 0.05). An association between ΔMTV (*p* = 0.037) and response to neo-CTX was observed, while other ΔPET parameters did not show significant associations (Table 4). No statistically significant associations have been observed between PET EORTC response to therapy criteria and the pathological response to neo-CTX. These results are summarized in Additional file 1: Table S3.

**Table 4:** [^18^F]FDG PET parameters and response to neoadjuvant CTX. ΔMTV parameter was significantly associated with pathological response to Neo-CTX (Mann–Whitney test). IQR: interquartile range

	Tissue Response to Neo-CTX ≤ 90%			Tissue Response to Neo-CTX > 90%			*p* value
	IQR 25	Median	IQR 75	IQR 25	Median	IQR 75	
PET1-SUVmax	6.0	8.4	10.6	5.1	7.9	11.2	0.949
PET1-SUVmean	2.4	4.0	4.8	2.9	4.5	5.1	0.652
PET1-SUVpeak	3.8	6.7	7.5	4.0	5.5	6.9	0.813
PET1-MTV	41.1	171.4	261.3	102.7	139.5	336.7	0.377
PET1-TLG	15.9	108.1	259.9	72.0	112.4	302.0	0.377
PET2-SUVmax	2.7	3.4	5.2	2.2	2.9	3.4	0.201
PET2-SUVmean	1.8	2.1	3.0	1.4	1.8	2.2	0.146
PET2-SUVpeak	2.3	2.8	4.5	1.8	1.9	2.9	0.146
PET2-MTV	54.7	101.7	176.1	34.8	67.2	103.4	0.310
PET2-TLG	31.7	44.3	172.1	19.4	46.1	80.6	0.683
Δ SUVmax	− 64%	− 57%	− 11%	− 85%	− 59%	− 36%	0.377
Δ SUVmean	− 51%	− 34%	− 3%	− 69%	− 50%	− 38%	0.102
Δ SUVpeak	− 62%	− 46%	− 11%	− 74%	− 58%	− 40%	0.234
Δ MTV	− 52%	− 34%	− 14%	− 87%	− 59%	− 35%	0.037
Δ TLG	− 60%	− 50%	− 22%	− 94%	− 63%	− 45%	0.051

### Bone sarcoma only subpopulation

Considering the presence of multiple tumor types in our pediatric population, a post hoc sub-analysis was performed in patients with bone sarcoma only (*n* = 26; osteosarcoma = 17; Ewing's sarcoma = 9). In this bone sarcoma subpopulation, all PET2 parameters showed a statistically significant association with TTP, confirming the results also observed in the full study population (Additional file 1: Table S4).

## Discussion

In this prospective single-center study, a significant association between early interim [^18^F]FDG PET2 parameters and TTP was observed. Patients with higher residual disease metabolic activity at 6 weeks after initiation of neo-CTX study had worse outcomes compared to those with no or only mild residual [^18^F]FDG activity. Additionally, patients showing a lower MTV reduction from PET1 to PET2 had a lower pathological response rate. However, none of the PET parameters were predictive of OS.

[^18^F]FDG PET/CT is important for staging and therapy response assessment of patients with high-grade bone and soft tissue sarcomas [10, 20]. However, limited data are currently available regarding the potential role of early interim PET/CT performed 6 weeks after neo-CTX initiation as a predictor of patient outcome, especially in the pediatric population. Conflicting published results are probably due to heterogeneous patient populations, different therapy regimen and different time points of PET/CT evaluations. Costelloe et al. reported in a mixed population of pediatric and adult bone sarcoma patients that SUVmax and TLG values measured before and after neo-CTX provided predictive information about treatment response [21]. In a small study of bone sarcoma patients, changes in [^18^F]FDG SUVmax at the end of neo-CTX predicted histopathologic responders and non-responders [22]. However, as both studies measured glucose metabolic parameters after completion of neo-CTX, the impact on managing these patients appears limited. In the present study, glucose metabolism responses were measured early during neo-CTX. Earlier identification of non-responders to neo-CTX could lead to meaningful treatment changes. In the current study, a significant association between the early interim [^18^F]FDG PET parameters and patient outcome was observed. However, no significant association between the baseline [^18^F]FDG PET parameters and TTP was observed. This contrasts with other studies reporting PET1-SUVmax as a prognostic biomarker [23, 24].

None of PET1, PET2 and ΔPET parameters were associated with OS. This is probably due to the relatively small sample size and the limited number of events in the current study population.

Histologic response to neo-CTX is known to be a prognostic indicator in bone and soft tissue sarcoma, especially in osteosarcoma. Patients with > 90% tumor necrosis in response to treatment have improved outcomes [13]. In contrast to other studies [24–27], we did not observe a significant association between any baseline or early interim [^18^F]FDG PET parameters and the response to treatment assessed by the percentage of tumor necrosis (*n* = 29) or by MRI evaluation (*n* = 5). However, we observed a significant association of ΔMTV with the pathological response to neo-CTX. Additionally, patients with higher PET2-SUVmax were less likely to be responders to neo-CTX although without statistical significance. These results suggest that tumor metabolic activity changes under neo-CTX as assessed on early interim [^18^F]FDG PET2 can be integrated into the clinical risk prognostic assessment.

[^18^F]FDG PET/CT is a whole-body imaging modality and can detect distant metastatic lesions. In our cohort, patients with metastatic disease had worst outcome. Of note, all metastatic lesions showed partial or complete response on PET2. It is considered standard of care to treat distant metastases with local control methods (surgery and/or radiation). Whether or not the prognosis is altered with this aggressive approach is controversial.

The main limitation of the study is its small sample size and the heterogeneity of the included sarcoma sub-types. However, each tumor sub-type was treated under the same therapy protocol. Results of the study were comparable in the subpopulation of patients with bone sarcoma only (osteo- and Ewing's sarcoma). Of note, patients with rhabdomyosarcoma were not included in this study as they were enrolled on competing COG therapeutic clinical trials, which included PET imaging as an experimental aim. Further sub-analyses considering the different tumor subtypes were not feasible considering the small sample size and the limited number of events. Larger and more homogeneous cohorts will be required to determine whether early interim [^18^F]FDG PET/CT imaging can be useful for early treatment response predictions and prognostic information. However, such studies are difficult to conduct as bone and soft tissue sarcomas are rare neoplasms, especially when only considering pediatrics patients only. Another limitation is the lack of control for tumor necrosis. The data reported were obtained using the pathology and MRI clinical reports considered as reference in the treatment management of the patient. Finally, the comparison with the RECIST criteria for assessing the response to neo-CTX was not performed because of heterogeneous conventional imaging follow-up (modality, time points).

## Conclusion

In this prospective single center study of 34 pediatric patients with sarcoma, the intensity of residual metabolic tumor activity on early interim [^18^F]FDG PET/CT studies performed 6 weeks after the start of neo-CTX was associated with earlier TTP but not OS. Additionally, MTV reduction after neo-CTX was associated with tumor pathological response. [^18^F]FDG PET/CT may serve as a useful early prognostic marker in pediatric patients with high-grade bone and soft tissue sarcoma.

## Supplementary information

**Additional file 1**.

## Data Availability

The datasets used during the current study are available from the corresponding author on reasonable request.
