# Supplementary material for: [18F]FDG PET/CT for evaluating early response to neoadjuvant chemotherapy in pediatric patients with sarcoma: a prospective single-center trial
Source: EJNMMI Res. 2020 Oct 15;10:122. doi: 10.1186/s13550-020-00715-0 (PMC7561652; doi:10.1186/s13550-020-00715-0)
Supplement: Supplementary file 1 — Additional file 1. [file 13550_2020_715_MOESM1_ESM.docx]

**Supplemental Figure 1**

Kaplan-Meier plot analyses of PET2-parameters with TTP. Patients were stratified by the median values for SUVpeak and SUVmean and by the upper quartile valure for MTV and TLG.


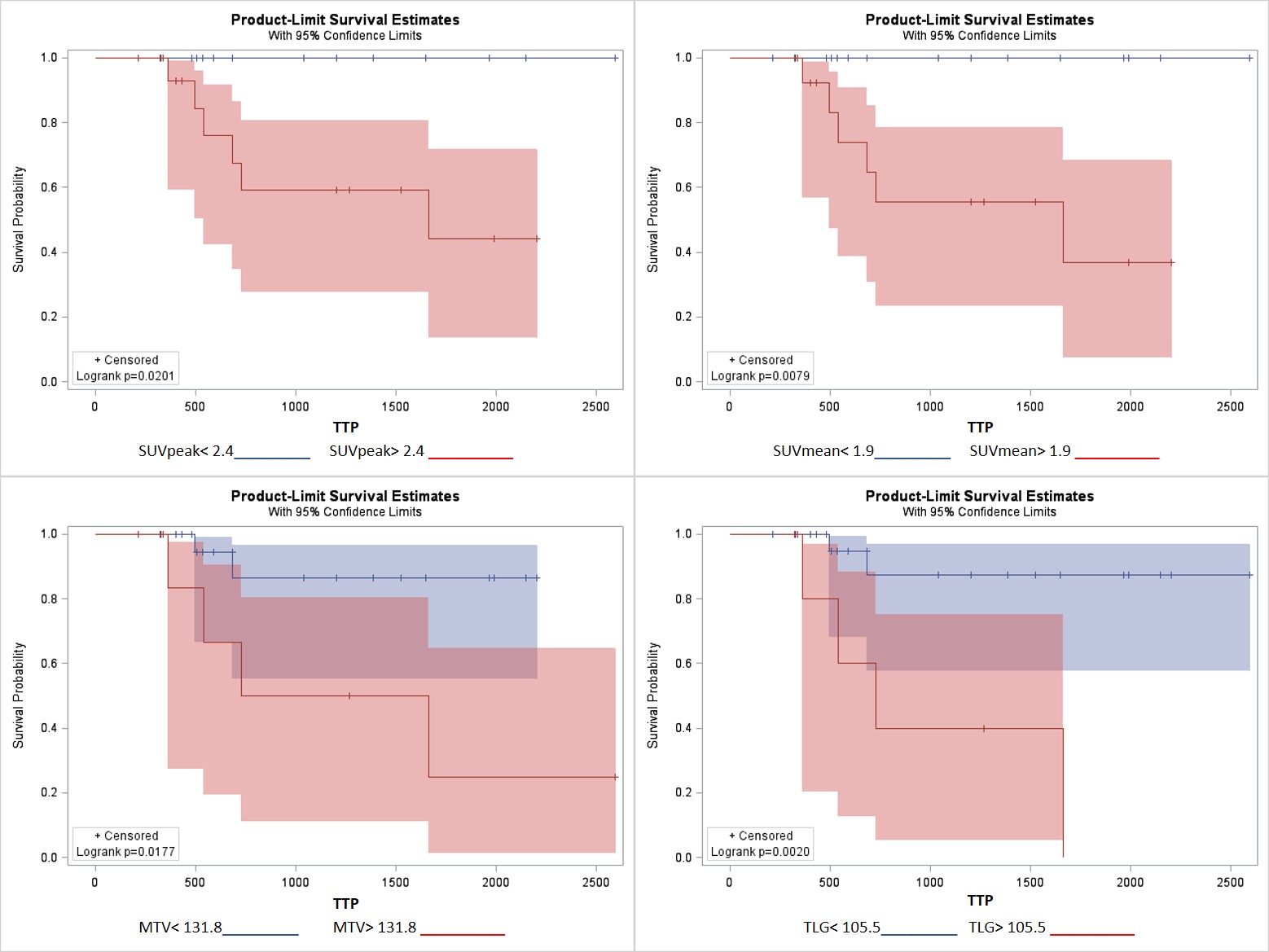


**Supplemental Table 1: ΔPET parameters and relapse status at last follow-up**

|  | **ΔSUVmax** | **ΔSUVmean** | **ΔSUVpeak** | **ΔMTV** | **ΔTLG** |
| --- | --- | --- | --- | --- | --- |
|  | *Median Value* | *Median Value* | *Median Value* | *Median Value* | *Median Value* |
| **No Relapse** | -68% | -51% | -64% | -66% | -69% |
| **Relapse** | -57% | -38% | -47% | -41% | -53% |
| **p value*** | 0.129 | 0.017 | 0.058 | 0.028 | 0.031 |
| * *Mann-Whitney test* | | | | | |

**Supplemental Table 2: [^18^F]FDG PET parameters and OS.**

Univariate analysis of PET1, PET2 and ΔPET for OS in the entire population (bone + soft tissue sarcoma)

|  | **HR** | **95,0% CI** | | **P value** |
| --- | --- | --- | --- | --- |
|  |  | **Lower** | **Upper** |  |
| **PET1-SUVmax** | 1.01 | 0.84 | 1.20 | 0.93 |
| **PET1-SUVmean** | 1.03 | 0.63 | 1.69 | 0.89 |
| **PET1-SUVpeak** | 0.98 | 0.79 | 1.26 | 0.98 |
| **PET1-MTV** | 0.99 | 0.99 | 1.01 | 0.53 |
| **PET1-TLG** | 0.99 | 0.99 | 1.01 | 0.61 |
|  |  |  |  |  |
| **PET2-SUVmax** | 1.05 | 0.89 | 1.23 | 0.56 |
| **PET2-SUVmean** | 1.50 | 0.88 | 2.58 | 0.137 |
| **PET2-SUVpeak** | 1.12 | 0.89 | 1.40 | 0.32 |
| **PET2-MTV** | 1.01 | 0.99 | 1.01 | 0.54 |
| **PET2-TLG** | 1.00 | 1.00 | 1.01 | 0.04 |
|  |  |  |  |  |
| **ΔSUVmax** | 1.00 | 0.98 | 1.02 | 0.48 |
| **ΔSUVmean** | 1.00 | 0.99 | 1.02 | 0.33 |
| **ΔSUVpeak** | 1.07 | 0.99 | 1.02 | 0.39 |
| **ΔMTV** | 1.00 | 0.99 | 1.01 | 0.30 |
| **ΔTLG** | 1.00 | 1.00 | 1.00 | 0.03 |

**Supplementary Table 3**

| **EORTC Criteria for PET response to neo-CTX** | **Tissue Response**  **to Neo-CTX** | | | ***p value*** |
| --- | --- | --- | --- | --- |
|  | Non-responder | Responder | Total |  |
| Complete Response (CR) | 1 | 0 | 1 | 0.6251 |
|  | 100% | 0% |  |  |
| Progressive Disease (PD) | 3 | 1 | 4 |  |
|  | 75% | 25% |  |  |
| Partial Response (PR) | 12 | 12 | 24 |  |
|  | 50% | 50% |  |  |
| Stable Disease (SD) | 3 | 2 | 5 |  |
|  | 60% | 40% |  |  |

**Supplementary Table 4: [^18^F]FDG PET2 parameters and TTP** in the sub-population of bone sarcoma only (n=26) (Univariate analysis).

|  | **HR** | **95,0% CI** | | **P value** |
| --- | --- | --- | --- | --- |
|  |  | **Lower** | **Upper** |  |
| **PET2-SUVmax** | 1.26 | 1.06 | 1.49 | 0.008 |
| **PET2-SUVmean** | 1.58 | 1.10 | 2.28 | 0.014 |
| **PET2-SUVpeak** | 1.33 | 1.06 | 1.66 | 0.012 |
| **PET2-MTV** | 1.00 | 1.00 | 1.01 | 0.033 |
| **PET2-TLG** | 1.00 | 1.00 | 1.01 | 0.027 |
